# Perception of the risk of tobacco use in pregnancy and factors associated with tobacco use in rural areas of Myanmar

**DOI:** 10.18332/tpc/112719

**Published:** 2019-10-24

**Authors:** Kyaw L. Show, Aung P. Phyo, Saw Saw, Ko K. Zaw, Thuzar C. Tin, Nyein A. Tun, Khin T. Wai

**Affiliations:** 1 Department of Medical Research, Ministry of Health and Sports, Yangon, Myanmar; 2 University of Public Health, Yangon, Myanmar; 3 Department of Public Health, Ministry of Health and Sports, Naypyitaw, Myanmar

**Keywords:** pregnancy, tobacco, rural, perception

## Abstract

**INTRODUCTION:**

Tobacco use is recognized as the most important preventable risk factor for pregnancy complications and undesirable fetal outcomes. This study examined the reported prevalence of tobacco use among married men and women residing in rural areas, and their knowledge on the risks of tobacco use during pregnancy and the factors associated with tobacco use.

**METHODS:**

A cross-sectional study was conducted within 32 villages in the delta region of Myanmar, randomly selected through multistage sampling procedure by using a pre-tested structured questionnaire during 2016. In all, 617 people participated in the household survey.

**RESULTS:**

About 80% of current smokers (109/128) smoked at home, of whom 16% reported the presence of a pregnant woman in their smoking area. Less than 25% of the respondents were aware of the negative impacts of tobacco use on pregnancy outcomes. Men had significantly lower perceived risk towards smoking on some pregnancy outcomes. Multivariate analysis confirmed the significant influence of male gender (adjusted OR, AOR=12.62; 95% CI: 6.30–25.29) and the age of women <35 years (AOR=3.51; 95% CI: 1.97–6.26) on current tobacco use, when controlling for other variables.

**CONCLUSIONS:**

Men in the study villages and those with a low level of education had poor knowledge on the risks of tobacco on pregnancy outcomes. However, good knowledge and perceived risk of undesirable impacts on pregnancy did not have any influence on tobacco use.

## INTRODUCTION

Tobacco use is a major public health concern globally. It is the most important preventable risk factor for pregnancy complications and dangerous fetal outcomes^1^; cigarette smoking poses a threat both to pregnant women and their newborns, such as tobacco-induced abortions and deaths from perinatal disorders. Also, smokeless tobacco use during pregnancy can have a higher risk of pregnancy complications with unfavorable fetal outcomes. Moreover, maternal exposure to secondhand smoke (SHS) in pregnancy may affect the birth-weight of newborns^2–5^. Studies from Asia and Sub-Saharan Africa indicate an increased risk of infant and child mortality due to tobacco smoke exposure^6,7^. Thus, several nations have given priority to the implementation of policies for the prevention of smoking in pregnancy^8-11^. The nationwide survey in 2009 informed of the higher prevalence of current daily smoking and current daily smokeless tobacco use in rural areas compared to urban settings. In Myanmar, the STEP survey in 2014 reported that approximately 26% of people (44% of men and 8% of women) were current smokers and about 43% (62% of men and 24% of women) were current users of smokeless tobacco^12^. In 2015, a Myanmar Demographic and Health Survey reported that one in 25 of women of reproductive age (15– 49 years) used either one of the tobacco products^13^. Among others, the Ayeyawady Region revealed a high prevalence of tobacco use. The regional prevalence estimates of current daily smoking and current daily smokeless tobacco use were 19.6% and 30.3%, respectively^14^. Furthermore, the Ayeyawady Region reported an infant mortality rate of 87 per 1000 live births, which ranked second in Myanmar in 2016 and indicated a need to ascertain the underlying causes^15^. The Ministry of Health and Sports (MoHS) of Myanmar has pointed to the dangers of tobacco use on pregnancy complications and unfavorable fetal outcomes through health information, education and communication materials^16^.

Risk perception is critical for the decision of smokers to quit smoking, as noted in several studies in developed countries^17-19^. However, there are no studies on the influence of knowledge and perceived risks towards the use of tobacco among adults in rural areas of Myanmar. This is an under-researched area in how people perceive the risks of harmful substances that may affect their use. Further knowledge on risk perception is required to design health promotion programs. The present study attempted to examine the reported prevalence of smoking and smokeless tobacco use among married men and women residing in rural areas, and their knowledge of the risks in pregnancy due to tobacco use. The overall aim was to provide evidence to inform public health programs on reducing the infant mortality rate through tobacco cessation as the essential strategy.

## METHODS

### Study design

We conducted a cross-sectional household study in 32 villages of Kyaunggon and Lemyethna townships in the Ayeyawady Region of Myanmar from August 2016 to July 2017. Administratively, the Republic of the Union of Myanmar is divided into seven states and seven regions and a Nay Pyi Taw Council Union Territory. Myanmar has an estimated population of 51.5 million according to the 2014 National Census^15^, with nearly 70% residing in rural areas. The Department of Public Health under the MoHS is responsible for providing primary health care services, including information, education and communication through rural health centers (RHC) and subcenters. The National Census Report 2014 also indicated that 86 in 100 persons from the Ayeyawady Region were rural residents. Altogether 1.1 million out of 1.6 million women aged 15–49 years (reproductive age group) in the Ayeyawady Region were ever married. The study population in the present research covered married women of reproductive age in the range 18– 49 years and their husbands.

### Sample size and sampling procedure

We assumed the anticipated population proportion of current smokers in Myanmar as 26% according to the STEP survey 2014^12^. Allowing for an error margin of 0.05 at the 95 % confidence interval and a 5% non-response rate, the required sample size was 320 respondents for married men and women, respectively, for a total of 640 respondents. A multistage sampling procedure was used. In the first stage, the research team purposively included two out of 33 townships in the Ayeyawady Region. Then, two rural health centers (RHCs) from each township were randomly selected. Next, under the jurisdiction of each RHC, a two-way stratification method was used to avoid selection bias and included four villages with Sub-RHC and four villages without Sub-RHC, randomly. This two-way stratification procedure allowed us to attain the unbiased estimates without a need to consider for design effect^20^. Finally, ten men and ten women per study site were selected at random to meet the required sample size. Owing to limited resources, the survey team was unable to meet the standard sample size of 20 per gender category in each site. Local administrative authorities provided the list of eligible households in selected sites. If the eligible person was not available during the household visit between 9 am and 5 pm, the team used the nearest eligible household. There were no callbacks during the field survey due to time constraints. In all, 617 out of 640 eligible respondents (96.4%) participated in the household survey. The non-response rate in this study only referred to refusals to participate in the survey.

### Data collection

Following written informed consent, trained interviewers administered a pre-tested structured questionnaire during face-to-face interviews with one eligible participant per selected household. The pre-tested and modified structured interview questionnaire covered four components: sociodemographic characteristics; awareness on the miscarriages and fetal outcomes due to tobacco (smoked tobacco, smokeless tobacco, and secondhand tobacco smoke) on pregnancy; their perceptions measured by three categorical items ‘yes’, ‘no’, and ‘do not know’; and practices. Current smokers and smokeless tobacco users were defined as the people who smoked cigarettes/used smokeless tobacco on a daily basis and occasionally. Only women were asked for perceived risks due to smokeless tobacco. Exposure to SHS was defined as tobacco smoke that was exhaled by a smoker and was inhaled by persons nearby.

### Data analysis

After checking for consistency and completeness, collected data were entered by Epi-data version 3.2 (EpiData Association, Denmark) and analyzed by IBM SPSS Statistics version 22.0 (SPSS, Inc., Chicago IL, USA). The reported prevalence rates and 95% CIs were computed. Accordingly, the level of knowledge as the first outcome measure of interest was categorized as ‘poor’ for respondents who scored ≤ mean value and ‘good’ for those who scored > mean value. We transformed a ‘yes’ response to perception questions of five possible pregnancy risks: miscarriages, preterm, low birth weight babies, stillbirth, and congenital anomalies into one composite measure of perception to smoking, smokeless tobacco or SHS exposure. Current smoking by both married men and women and current smokeless tobacco use (only for women) were additional outcome measures that were dichotomized into ‘yes’ and ‘no’ categories. For cross-tabulations, the chi-squared test was used to assess the contribution of sociodemographic characteristics on differences in outcome measures and a p<0.05 was considered statistically significant. Simple logistic regressions were performed to determine the factors associated with the levels of knowledge scores and current tobacco use when adjusted for confounders.

We obtained ethics approval from Ethics Review Committee, Department of Medical Research, Ministry of Health and Sports, Myanmar (Ethics/ DMR/2016/088, dated 4 July 2016). Privacy, confidentiality and anonymity issues were taken into account according to the Helsinki Declaration.

## RESULTS

The average age of the respondents was 35.6 years (95% CI: 35.0–36.2) and nearly 80% had low formal education (middle school level education and below). Four in five respondents were employed and more than half had a monthly family income of more than 100000 Kyat (approximately US$80). One in five respondents was a current smoker and cheroots predominated as the most commonly used product (a little cigar-like tobacco product). Males were significantly more likely than females to smoke cigarettes/cheroots (38.5% vs 3.8%) and use smokeless tobacco (73.1% vs 23.4%). The majority of current smokers (85.2%) smoked at home and 16.4% reported that there was a pregnant woman in their smoking area. Nearly half of the respondents currently used smokeless tobacco daily.

Table 1 presents the knowledge of the participants with regard to pregnancy outcomes due to tobacco use during pregnancy. The respondents cited low birth weight babies as the commonest consequence of smoking followed by pre-term labor and miscarriages. Married women mentioned the negative outcomes of pregnancy due to tobacco use more specifically than married men.

**Table 1:** Knowledge on the risks of tobacco use on pregnancy outcomes among married men and women, 32 villages, Ayeyawady Region, 2016

KNOWLEDGE*	*Total (n=617) n (%)*	*Male (n=301) n (%)*	*Female (n=316) n (%)*	*p*
**Knowledge on the risk of smoking on pregnancy outcomes**				
Abortion	88 (14.3)	33 (11.0)	55 (17.4)	0.022
Pre-term	124 (20.1)	41 (13.6)	83 (26.3)	<0.001
LBW baby	145 (23.5)	46 (15.3)	99 (31.3)	<0.001
Stillbirth	24 (3.9)	8 (2.7)	16 (5.1)	0.122
Congenital anomalies	50 (8.1)	20 (6.6)	30 (9.5)	0.195
**Knowledge on the risk of secondhand smoke exposure on pregnancy outcomes**				
Abortion	63 (10.2)	23 (7.6)	40 (12.7)	0.040
Pre-term	124 (20.1)	46 (15.3)	78 (24.7)	0.004
LBW baby	135 (21.9)	43 (14.3)	92 (29.1)	<0.001
Stillbirth	12 (1.9)	5 (1.7)	7 (2.2)	0.618
Congenital anomalies	37 (6.0)	15 (5.0)	22 (7.0)	0.301
**Knowledge on the risk of smokeless tobacco use on pregnancy outcomes**				
Abortion	49 (7.9)	18 (6.0)	31 (9.8)	0.079
Pre-term	116 (18.8)	37 (12.3)	79 (25.0)	<0.001
LBW baby	139 (22.5)	42 (14.0)	97 (30.7)	<0.001
Stillbirth	15 (2.4)	5 (1.7)	10 (3.2)	0.226
Congenital anomalies	28 (4.5)	11 (3.7)	17 (5.4)	0.303

* Single item positive responses only. LBW: low birth weight.

According to Table 2, about 65% to 86% of men and 71% to 90% of women expressed their perceived risks of smoking on miscarriages and fetal outcomes. About 60% to 80% of married men and married women had perceived risks of SHS exposure on miscarriages and fetal outcomes. Moreover, 53% and 73% of married women perceived the risks of miscarriages and fetal outcomes, respectively, when they used smokeless tobacco.

**Table 2:** Perceived risks of tobacco use on pregnancy outcomes among married men and women, 32 villages, Ayeyawady Region, 2016

PERCEPTIONS*	*Total (n=617) n (%)*	*Male (n=301) n (%)*	*Female (n=316) n (%)*	*p*
**Perceived risk of smoking on pregnancy outcomes**				
Abortion	428 (69.4)	197 (65.4)	231 (73.1)	0.039
Pre-term	494 (80.1)	229 (76.1)	265 (83.9)	0.016
LBW baby	526 (86.9)	252 (83.7)	284 (89.9)	0.024
Stillbirth	429 (69.5)	206 (68.4)	223 (70.6)	0.565
Congenital anomalies	532 (86.2)	259 (86.0)	273 (86.4)	0.901
**Perceived risk of exposure to secondhand smoke on pregnancy outcomes**				
Abortion	392 (63.5)	195 (64.8)	197 (62.3)	0.529
Pre-term	454 (73.6)	224 (74.4)	230 (72.8)	0.645
LBW baby	492 (79.7)	237 (78.7)	255 (80.7)	0.545
Stillbirth	375 (60.8)	187 (62.1)	188 (59.5)	0.503
Congenital anomalies	488 (79.1)	244 (81.1)	244 (77.2)	0.240
**Perceived risk of smokeless tobacco use on pregnancy outcomes**				
Abortion	-	-	169 (53.5)	-
Pre-term	-	-	208 (65.8)	-
LBW baby	-	-	220 (69.6)	-
Stillbirth	-	-	170 (53.8)	-
Congenital anomalies	-	-	231 (73.1)	-

* Single item positive responses only. LBW: low birth weight.

Table 3 elucidates the factors associated with knowledge concerning the risk of tobacco use on pregnancy outcomes. Multivariate analysis confirmed that being male (AOR=2.96, 95% CI: 2.05–4.26) was significantly associated with the lack of knowledge of any possible risk of smoking and SHS exposure. Moreover, those who had a higher formal education were more likely to be aware of the risks of smoking to pregnancy, however this difference was not evident for exposure to SHS or the use of smokeless tobacco.

**Table 3:** Knowledge on the risks of tobacco use on pregnancy outcomes and associated factors among married men and women, 32 villages, Ayeyawady Region, 2016

*Variables*	Smoking*			Secondhand smoke exposure*			Smokeless tobacco use* (for females only)		
	*n (%)*	*crude OR*	*AOR*	*n (%)*	*crude OR*	*AOR*	*n (%)*	*crude OR*	*AOR*
**Gender**									
Married men (n=301)	206 (68.4)	2.91 (2.09–4.04)	2.96 (2.05–4.26)	212 (70.4)	2.05 (1.47–2.85)	2.25 (1.56–3.24)	NA	NA	NA
Married women (n=316)	135 (42.7)	Ref.	Ref.	170 (53.8)	Ref.	Ref.			
**Age group** (years)									
>35 (n=314)	184 (58.6)	1.32 (0.96–1.81)	1.21 (0.87–1.69)	202 (64.3)	1.23 (0.89–1.71)	1.18 (0.84–1.65)	86 (56.2)	1.33 (0.86–2.07)	1.34 (0.85–2.10)
18–35 (n=303)	157 (51.8)	Ref.	Ref.	180 (59.4)	Ref.	Ref.	80 (49.1)	Ref.	Ref.
**Education**									
≤ Middle school (n=473)	276 (58.4)	1.70 (1.17–2.48)	1.73 (1.17–2.57)	298 (63.0)	1.22 (0.83–1.78)	1.27 (0.86–1.88)	133 (55.2)	1.57 (0.93–2.64)	1.65 (0.96–2.84)
≥ High school (n=144)	65 (45.1)	Ref.	Ref.	84 (58.3)	Ref.	Ref.	33 (44.0)	Ref.	Ref.
**Occupation**									
Employed (n=510)	295 (57.8)	1.82 (1.19–2.77)	0.96 (0.60–1.55)	319 (62.5)	1.17 (0.76–1.78)	0.74 (0.46–1.19)	107 (50.5)	0.78 (0.49–1.25)	0.72 (0.44–1.17)
Dependant (n=107)	46 (43.0)	Ref.	Ref.	63 (58.9)	Ref.	Ref.	59 (56.7)	Ref.	Ref.
**Family income** (Kyat)									
									
>100000 (n=339)	187 (55.2)	0.99 (0.72–1.36)	1.07 (0.76–1.49)	218 (64.3)	1.25 (1.90–1.74)	1.29 (0.92–1.81)	94 (53.1)	1.05 (0.67–1.64)	1.12 (0.71–1.79)
≤100000 (n=278)	154 (55.4)	Ref.		Ref.	164 (59.0)	Ref.	Ref.	72 (51.8)	Ref.

* Do not know at least one of five possible pregnancy risks: miscarriages, preterm, low birth weight babies, stillbirth, and congenital anomalies. AOR: adjusted odds ratio. Exchange rate: 1000 Kyat about 0.8US$.

According to Table 4, it was significantly common for married men to smoke (AOR=12.62; 95% CI: 6.30– 25.29) compared to married women, when controlling for other variables. Other sociodemographic differentials, knowledge and risk perception did not have a significant influence on smoking practice.

**Table 4:** Current tobacco use and its associated factors among married men and women, 32 villages, Ayeyawady Region, 2016

*Variables*	*Current smoker*		
	*n (%)*	*Crude OR*	*AOR*
**Gender**			
Married women (n=316)	12 (3.8)	Ref.	Ref.
Married men (n=301)	116 (38.5)	15.86 (8.53–29.58)	12.62 (6.30–25.29)
**Age group** (years)			
18–35 (n=303)	52 (17.2)	Ref.	Ref.
>35 (n=314)	76 (24.2)	1.54 (1.04–2.29)	1.51 (0.96–2.36)
**Education**			
≥ High school (n=144)	29 (20.1)	Ref.	Ref.
≤ Middle school (n=473)	99 (20.9)	1.05 (0.66–1.67)	0.91 (0.53–1.55)
**Occupation**			
Dependent (n=107)	4 (3.7)	Ref.	Ref.
Employed (n=510)	124 (24.3)	8.27 (2.98–22.92)	1.41 (0.44–4.55)
**Family income** (Kyat)			
>100000 (n=339)	65 (19.2)	Ref.	Ref.
≤100000 (n=278)	63 (22.7)	1.24 (0.84–1.82)	1.33 (0.85–2.07)
**Known pregnancy risk due to smoking**			
At least one (n=276)	32 (11.6)	Ref.	Ref.
None (n=341)	96 (28.2)	2.99 (1.93–4.63)	1.62 (0.85–3.08)
**Known pregnancy risk due to secondhand smoke exposure**			
At least one (n=235)	30 (12.8)	Ref.	Ref.
None (n=382)	98 (25.7)	2.36 (1.51–3.69)	1.27 (0.65–2.47)
**Perceived risk of smoking to pregnancy**			
Good perception (n=436)	80 (18.3)	Ref.	Ref.
Poor perception (n=181)	48 (26.5)	1.61 (1.07–2.42)	1.49 (0.82–2.69)
**Perceived risk of secondhand smoke exposure to pregnancy**			
Good perception (n=386)	78 (20.2)	Ref.	Ref.
Poor perception (n=231)	50 (21.6)	1.09 (0.73–1.63)	0.83 (0.46–1.49)

Exchange rate: 1000 Kyat about 0.8US$. AOR: adjusted odds ratio.

Table 5 analyzes factors associated with smokeless tobacco use among a subset of married women (n=316). Those in the older age group, i.e. aged >35 years, significantly reported the use of smokeless tobacco (AOR=3.51; 95% CI: 1.97–6.26) compared to younger married women.

**Table 5:** Smokeless tobacco practice and its associated factors among married women, 32 villages, Ayeyawady Region, 2016

*Variables*	*Current smokeless tobacco user*		
	*n (%)*	*Crude OR*	*AOR*
**Age group** (years)			
18–35 (n=163)	22 (13.5)	Ref.	Ref.
>35 (n=153)	53 (34.6)	3.39 (1.94–5.94)	3.51 (1.97–6.26)
**Education**			
≥ High school (n=75)	12 (16.0)	Ref.	Ref.
≤ Middle school (n=241)	63 (26.1)	1.86 (0.94–3.67)	1.55 (0.75–3.22)
**Occupation**			
Dependent (n=104)	18 (17.3)	Ref.	Ref.
Employed (n=212)	57 (26.9)	1.76 (0.97–3.18)	1.43 (0.76–2.67)
**Family income** (Kyat)			
>100000 (n=177)	36 (20.3)	Ref.	Ref.
≤100000 (n=139)	39 (28.1)	1.53 (0.91–2.57)	1.52 (0.87–2.67)
**Known pregnancy risk due to smokeless tobacco**			
None (n=166)	36 (21.7)	Ref.	Ref.
At least one (n=150)	39 (26.0)	1.27 (0.76–2.13)	1.37 (0.78–2.41)
**Perceived risk of smokeless tobacco to pregnancy**			
Poor perception (n=147)	31 (21.1)	Ref.	Ref.
Good perception (n=169)	44 (26.0)	1.32 (0.78–2.23)	1.25 (0.71–2.19)

Exchange rate: 1000 Kyat about 0.8US$. AOR: adjusted odds ratio

## DISCUSSION

To the best of our knowledge, this is the first study in Myanmar that specifically explored the knowledge and risk perceptions and associated factors of tobacco use among married men and women. There was a moderate degree of reported smoking and the use of smokeless tobacco, especially among men who were married to women of reproductive age in rural areas of the Ayeyawady Region. The prevalence of using tobacco (both smoked and smokeless forms) ranged between 21% and 48%, respectively, among married men and women (combined) in rural areas. One study in the Yangon Region in Myanmar reported that the prevalence of smokeless tobacco use in rural households was approximately 38%^21^. The overall reported prevalences in this study were similar to those of the nationwide STEP survey report (2014), 20.7% vs 26.1% current smokers, and 47.6% vs 43.2% currently using smokeless tobacco^12^. However, a meta-analysis study performed in Myanmar by Kyaing et al.^22^ in 2012 reported a lower prevalence of 20.8% compared to our study. Even though different surveys used different data collection methods, the reported prevalence of tobacco use did not vary widely. Furthermore, the results clearly indicate that tobacco use was predominant among males in all studies mentioned above. In 2012, the prevalence estimates of current smokers was 46% of males and 5% of females among ASEAN countries, and 37% of men and 7% of women globally^23,24^. Even though smokeless tobacco practice was popular among men in most South-East Asian countries, women were more likely to use smokeless tobacco in Bangladesh, Indonesia and Thailand^25,26^ and the reverse was true in the present study among married people in rural areas.

It is well documented that tobacco use in either smoked or smokeless form can harm not only the pregnancy but also the fetus^3,5,8,27,28^. However, in this study, less than 25% (lowest of 1.9% to highest of 23.5%) of respondents in rural areas mentioned the negative impact of tobacco use on pregnancy outcomes, which was unsatisfactory. Addressing knowledge of smoking risks and cessation counseling in indigenous communities is a priority for antenatal programs^29^. Married men had significantly lower knowledge of the negative impact of tobacco use on pregnancy outcomes compared to women. In addition, low formal education had a significant influence on poor knowledge. This finding highlights the necessity to seek more effective channels to convey health information to married people from rural areas on risks of tobacco on miscarriages and fetal outcomes.

### Limitations

Despite the fact that women could express at a better level than men the perceived risks on the effects of smoking on miscarriages and fetal outcomes, the influence of knowledge and risk perception on tobacco use was not significant in this study. This might be due to underreporting of tobacco use or the sample size was small. Besides, under-reporting of tobacco use among female respondents was more likely, especially cigarette smoking, compared to men.

This study was conducted in 32 villages in two townships of the Ayeyawady Region and hence cannot be generalized to all rural married people of Myanmar. Above all, there was the possibility of social desirability bias related to positive responses towards knowledge and risk perceptions.

## CONCLUSIONS

Translation of evidence-based findings and strengthening knowledge transfer mechanisms of correct information to prevent tobacco exposure in pregnancy through effective risk communication programs is critical in promoting maternal and newborn health^5,30^. Conversely, concerted efforts to engage stakeholders are essential to introduce cost-effective prevention strategies to mitigate the negative impact of tobacco on pregnancy outcomes in a developing country like Myanmar. Men in study villages and those with a low level of education had poor knowledge on the risks of tobacco on pregnancy outcomes. However, good knowledge and perceived risk of undesirable impacts in pregnancy did not have any influence on tobacco use. Nevertheless, intensification of targeted health promotion programs by innovative approaches in rural areas, alongside tobacco regulations, is indispensable to reduce the prevalence of both smoke and smokeless tobacco use.
